# Y‐Box‐Binding Protein 1 Facilitates the Proliferation and Osteogenic Differentiation of Periodontal Ligament Stem Cells Through the Transcriptional Activation of FGF2‐Mediated Akt/GSK3β/β‐Catenin Signaling

**DOI:** 10.1002/kjm2.70079

**Published:** 2025-07-30

**Authors:** Yun‐Hao Xi, Chang‐Shun Li, Pin‐Lin Wu, Xiao‐Yang Zhou, Qian‐Wen Li, Cheng‐Hui Shen

**Affiliations:** ^1^ Department of Stomatology Affiliated Hospital of Integrated Traditional Chinese and Western Medicine, Nanjing University of Chinese Medicine Nanjing China; ^2^ Department of Stomatology Jiangsu Province Academy of Traditional Chinese Medicine Nanjing China; ^3^ Department of Stomatology The Affiliated Stomatological Hospital of Xuzhou Medical University Xuzhou China; ^4^ Department of Orthopedics and Traumatology Affiliated Hospital of Integrated Traditional Chinese and Western Medicine, Nanjing University of Chinese Medicine Nanjing China

**Keywords:** fibroblast growth factor 2, osteogenic differentiation, periodontal ligament stem cells, periodontitis, Y‐box binding protein 1

## Abstract

Periodontal ligament stem cells (PDLSCs) are derived from periodontal tissue and can differentiate into osteoblasts, which are ideal materials for alveolar bone repair and periodontal tissue regeneration. In this study, we aimed to explore the effects of Y‐box binding protein 1 (YB‐1) on the osteogenic differentiation and proliferation of PDLSCs and its underlying mechanism. hPDLSC proliferation was detected by CCK‐8 and EdU assays. The osteogenic differentiation of hPDLSCs was clarified using alkaline phosphatase (ALP) activity detection and Alizarin red S (ARS) staining. The expression levels of osteogenic differentiation‐related factors, Akt/GSK3β/β‐catenin pathway‐related factors, YB‐1, and fibroblast growth factor 2 (FGF2) were evaluated using qPCR and Western blotting. The interplay between YB‐1 and FGF2 was clarified using ChIP and dual‐luciferase reporter gene assays. YB‐1 expression was markedly decreased in periodontitis clinical tissues but increased in hPDLSCs during osteogenic differentiation. Moreover, silencing YB‐1 suppressed the osteogenic differentiation and proliferation of hPDLSCs. In addition, YB‐1 promotes hPDLSC proliferation and osteogenic differentiation in a manner dependent on the activation of the Akt/GSK3β/β‐catenin signaling pathway, which is mediated by FGF2 transcriptional activation. Furthermore, the inhibitory effects of YB‐1 knockdown on the osteogenic differentiation and proliferation of hPDLSCs were antagonized by human recombinant FGF2. Taken together, our findings revealed that YB‐1 facilitates hPDLSC proliferation and osteogenic differentiation through increasing the level of FGF2 via transcriptional regulation, thereby activating the Akt/GSK3β/β‐catenin pathway.

**Abbreviations**
ALP: alkaline phosphatase
ARS: Alizarin red S
CCK8: Cell Counting Kit‐8
ChIP: chromatin immunoprecipitation assay
CST: cell signaling technology
DMEM: Dulbecco's modified Eagle medium
DPSCs: dental pulp stem cells
ECL: enhanced chemiluminescence
EdU: 5‐ethynyl‐2′‐deoxyuridine
ELISA: enzyme‐linked immunosorbent assay
FGF2: fibroblast growth factor 2
MSCs: mesenchymal stem cells
OCN: osteocalcin
OPN: osteopontin
PDL: periodontal ligament
PDLSCs: periodontal ligament stem cells
PVDF: polyvinylidene fluoride
qPCR: quantitative real‐time polymerase chain reaction
SDS–PAGE: sodium dodecyl sulfate–polyacrylamide gel electrophoresis
YB‐1: Y‐box‐binding protein 1

## 1 Introduction

Periodontitis is a chronic inflammatory disease characterized by the continuous stimulation of pathogenic bacteria and endotoxins to cause a host immune inflammatory response, resulting in the loss and damage of periodontal supporting tissues and ultimately toothlessness [1]. Therefore, the restoration of lost periodontal tissue is highly important for the treatment of periodontitis. Periodontal ligament stem cells (PDLSCs) are important cellular components in periodontal tissue that have great proliferation and multidifferentiation potential and can be differentiated into osteoblasts, odontoblasts, and fibroblasts [2]. Periodontal stem cells not only maintain the stability of the periodontal environment under normal conditions but also play prominent roles in the self‐renewal, regeneration, and repair of periodontal tissues [3, 4, 5, 6]. Therefore, a thorough understanding of the regulatory mechanisms of PDLSC proliferation and osteogenic differentiation is crucial for the treatment of periodontitis.

A variety of classical signaling pathways, such as the PI3K/AKT [7], p38 MAPK [8] and Wnt/β‐catenin [9] pathways, are involved in regulating the osteogenic differentiation of stem cells. Studies have shown that decreased AKT expression prevents osteoblasts from developing to maturity [10]. In addition, AKT is closely linked to the Wnt/β‐catenin signaling pathway during osteogenic differentiation, which can further activate β‐catenin by inhibiting GSK3β [11], and phosphorylated AKT and phosphorylated GSK3β can prevent the degradation of β‐catenin and effectively promote its nuclear translocation [12]. Studies have shown that electromagnetic fields can modulate the osteogenic differentiation of mesenchymal stem cells (MSCs) by activating the AKT signaling axis, thereby positively regulating bone remodeling [13]. Jia et al. reported that PSAT1 positively regulates the osteogenic differentiation of PDLSCs by the Akt/GSK3β/β‐catenin axis [14]. Therefore, exploring the factors regulating the Akt/GSK3β/β‐catenin signaling pathway for the osteogenic differentiation of PDLSCs is important.

Y‐box binding protein‐1 (YB‐1) is a member of the Y‐box binding protein family and consists of an amino N‐terminus, a cold shock domain (CSD) and a hydrophilic C‐terminus [15]. As a transcription factor and multifunctional protein, YB‐1 is widely found in both prokaryotes and eukaryotes [16] and plays a role in binding DNA and RNA [17, 18] as well as binding to the Y‐box sequence of the promoter, thus performing a variety of biological functions, such as transcriptional regulation and interactions with other proteins [19, 20]. In recent years, YB‐1 has been shown to play a biological role not only in tumor and organ fibrosis but also in regulating stem cell differentiation. For example, studies have shown that YB‐1 can facilitate the osteogenic differentiation of MSCs [21, 22]. However, the function and mechanism of YB‐1 in the osteogenic differentiation and proliferation of PDLSCs remain uncertain.

In the present study, we investigated the specific mechanism by which YB‐1 affects the osteogenic differentiation and proliferation of hPDLSCs. We hypothesized that YB‐1 was gradually upregulated during the differentiation of hPDLSCs and that the knockdown of YB‐1 inhibited the osteogenic differentiation and proliferation of hPDLSCs by transcriptional inactivation of the FGF2‐mediated AKT/GSK3β/β‐catenin signaling pathway.

## 2 Materials and Methods

### 2.1 Patients and Tissue Collection

A total of 12 periodontal ligament (PDL) tissues derived from the extracted third molars were collected from patients with severe periodontitis admitted to the hospital from October 2020 to December 2022 (the patients were aged 25–35 years and had no dental or periodontal diseases, no systemic diseases, and did not smoke). A total of 12 healthy PDL tissues from third molars extracted due to orthodontic treatment were taken as controls (the volunteers were 12–20 years old and were systemically healthy, had no periodontitis, and did not smoke). This research was reviewed and approved by the Ethics Committee of the hospital, and all patients provided signed informed consent.

### 2.2 hPDLSC Isolation and Culture

The PDLSCs were isolated as previously described [23]. In brief, PDL tissues from third molars were collected from healthy volunteers via orthodontic treatment at our hospital. After extraction, the PDL tissue was separated into 1 × 1 mm tissue blocks, which were placed on the bottom of the culture bottle, and the medium containing 20% fetal bovine serum (FBS) was replaced every 3 days. On day 7, the cells could be observed migrating from the tissue. After culture at a ratio of 1:2, the cell morphology was uniform and had a slender spindle shape. The PDLSCs from the 2nd to 5th generations were used for the experiments.

### 2.3 Immunophenotype Assay

To analyze the immune phenotype of PDLSCs, a BD Human MSC Analysis Kit (BD Biosciences, USA) was used according to the manufacturer's instructions. In brief, the cells were harvested and incubated with antibodies conjugated with fluorescent dyes in the dark at 4°C for 30 min. Then, the cells were washed with phosphate‐buffered saline (PBS) and detected by flow cytometry. The antibodies used in the kit included MSC‐positive markers (CD90, CD105, CD73, and CD44) and MSC‐negative markers (CD34, CD11, CD19, CD45, and HLA‐DR).

### 2.4 Cell Transfection and Treatment

Short hairpin‐targeting YB‐1 (sh‐YB‐1), FGF2 (sh‐FGF2) and YB‐1‐overexpressing (pc‐YB‐1) plasmids were obtained from GenePharma (Shanghai, China), while the corresponding negative controls were sh‐NC and pc‐NC, respectively. The sequences of the oligonucleotides used were as follows: sh‐YB‐1 (5′‐GATCCCCGTACCTTCGCAGTGTAGGATTCAAGAGATCCTACACTGCGAAGGTACTTTTTAGCTAAAAAGTACCTTCGCAGTGTAGGATCTCTTGAATCCTACACTGCGAAGGTACGGG‐3′); sh‐FGF2 (5′‐CCGGTATAGCTCAGTTTGGATAATTCTCGAGAATTATCCAAACTGAGCTATATTTTTG‐3′). Similarly, hPDLSCs were seeded onto 6‐well plates and allowed to grow until they reached approximately 80% confluence. The above plasmids were transfected into hPDLSCs utilizing Lipofectamine 3000 (Invitrogen, USA) in accordance with the instructions. After 48 h, qPCR was applied to analyze the transfection efficiency. To study the impact of the Akt pathway or FGF2 on YB‐1‐mediated osteogenic differentiation, hPDLSCs were treated with 1 μM Akt inhibitor (MK‐2206) or 50 ng/mL human recombinant FGF2 (FGF2) for 24 h.

### 2.5 Osteogenic Differentiation Induction

PDLSCs were inoculated into the culture plate, and after 12 h of cell adhesion, the medium was changed to osteogenic induction medium (containing 50 mg/L ascorbic acid, 10 nM dexamethasone, and 10 mM sodium β‐glycerophosphate), and the medium was changed every 2–3 days.

### 2.6 ALP Activity Detection

On the 7th day after the induction of osteogenic differentiation in PDLSCs, an ALP activity assay kit (Nanjing Jiancheng Bioengineering Institute, China) was used to measure ALP activity according to the manufacturer's instructions. The absorbance at a wavelength of 520 nm was tested with a microplate reader (Bio‐Rad, Hercules, CA).

### 2.7 Alizarin Red Staining

On the 21st day after osteogenic differentiation, the PDLSCs were stained with 1% alizarin red solution for 10 min, and the calcium nodules were observed and photographed.

### 2.8 Quantitative Real‐Time Polymerase Chain Reaction (qPCR)

TRIzol (Invitrogen, CA, USA) was used to harvest total RNA from tissues or cells, and cDNA was synthesized using a reverse transcription kit in accordance with the manufacturer's instructions. Thereafter, qPCR was carried out with BRYT Green Master Mix (Promega). The primer sequences used were as follows: YB‐1 (F): 5′‐GCAGGAGAACAAGGTAGACCAG‐3′; YB‐1 (R): 5′‐CTTCATTGCCGTCCTCTCTAGG‐3′; FGF2 (F): 5′‐AGCGGCTGTACTGCAAAAACGG‐3′; FGF2 (R): 5′‐CCTTTGATA GACACAACTCCTCTC‐3′; β‐actin (F): 5′‐GACATGGAGAAGATCTGGCA‐3′; and β‐actin (R): 5′‐GGTCTTTACGGATGTCAACG‐3′. β‐actin was utilized as the internal control, and the relative expression level of each gene was calculated by the 2^−ΔΔCt^ method.

### 2.9 Western Blotting

Total protein was acquired from tissues and cells utilizing RIPA lysis buffer (Beyotime, China). After quantification by a BCA protein assay (Solarbio, China), the same amount of protein was transferred to a polyvinylidene fluoride (PVDF) membrane (Millipore, Billerica, MA) by 10% sodium dodecyl sulfate–polyacrylamide gel electrophoresis (SDS–PAGE). Afterward, the membranes were incubated in 5% nonfat milk for 1 h and then incubated with the following primary antibodies: YB‐1 (ab76149, 1:1000, Abcam), FGF2 (ab208687, 1:1000, Abcam), RUNX2 (ab76956, 1:1000, Abcam), OCN (ab133612, 1:1000, Abcam), OPN (ab214050, 1:1000, Abcam), anti‐phospho‐Akt (p‐Akt) (#9271, 1:1000, CST), anti‐Akt (#4691, 1:1000, CST), anti‐p‐GSK‐3β (#25462, 1:1000, ST), anti‐GSK‐3β (#12456, 1:1000, CST), active β‐catenin (#19807, 1:1000, CST), β‐catenin (#8480, 1:1000, CST) and anti‐β‐actin (ab8226, 1:1000, Abcam) overnight at 4°C. Then, the membrane was incubated with an HRP‐labeled secondary antibody (ab205718, 1:5000, Abcam) for 2 h, and the protein bands were measured using an enhanced chemiluminescence (ECL) kit. The results were imaged and analyzed using ImageJ software.

### 2.10 Cell Counting Kit‐8 (CCK8) Assay

hPDLSCs were inoculated in 96‐well plates and cultured in an incubator at 37°C for 48 h. After the indicated treatment, 10 μL of CCK‐8 reagent (KeyGEN, China) was added to the cells, which were incubated at 37°C for 4 h. Finally, a microplate reader (Bio‐Rad, Hercules, CA) was used to measure the absorbance at 450 nm.

### 2.11 5‐Ethynyl‐2′‐Deoxyuridine (EDU) Detection

hPDLSCs were seeded onto 96‐well plates at a density of 5 × 10^5^ cells/well. After the indicated treatment, 50 μmol/L EdU was added and incubated at 37°C for 2 h. After fixation, the formaldehyde mixture was treated with Triton X‐100 and DAPI and observed by fluorescence microscopy. The percentage of EdU‐positive cells was calculated.

### 2.12 Chromatin Immunoprecipitation (ChIP) Assay

A Pierce Magnetic ChIP Kit (Thermo Fisher Scientific, Rockford, IL, USA) was used to validate the interaction between YB‐1 and FGF2. In brief, hPDLSCs were crosslinked with 1% formaldehyde at RT for 10 min, after which the crosslinking reaction was blocked with glycine. Next, the cell lysates were treated with Dynabeads conjugated with anti‐YB‐1 (ab76149, Abcam) at 4°C for 2 h. Subsequently, the beads were washed with IP wash buffer and eluted with elution buffer to decrosslink the DNA‐protein composites at 60°C for 2 h. Finally, qPCR was used to detect the purified DNA.

### 2.13 Dual‐Luciferase Reporter Gene Assay

The 3′‐UTR of the wild‐type or mutated fragment of FGF2 was inserted into the pGL3‐basic vector (Promega, USA). Then, pGL3 vectors containing the FGF2 promoter were cotransfected with pc‐NC or pc‐YB‐1 into hPDLSCs with Lipofectamine 3000. After transfection, a dual‐luciferase reporter assay system (Promega, USA) was used to examine the luciferase activity.

### 2.14 Statistical Analysis

All the experiments were repeated at least three times, and all the data were analyzed using GraphPad Prism 6.0 (GraphPad Prism; La Jolla, CA, USA) and are presented as the means ± standard deviations (SDs). Comparisons between two groups were performed using Student's *t* test, and comparisons among multiple groups were conducted utilizing one‐way ANOVA followed by Tukey's post hoc test. *p* < 0.05 was considered a statistically significant difference.

## 3 Results

### 3.1 Expression of YB‐1 in Clinical Tissues From Periodontitis Patients and hPDLSCs During Osteogenic Differentiation

**FIGURE 1:**
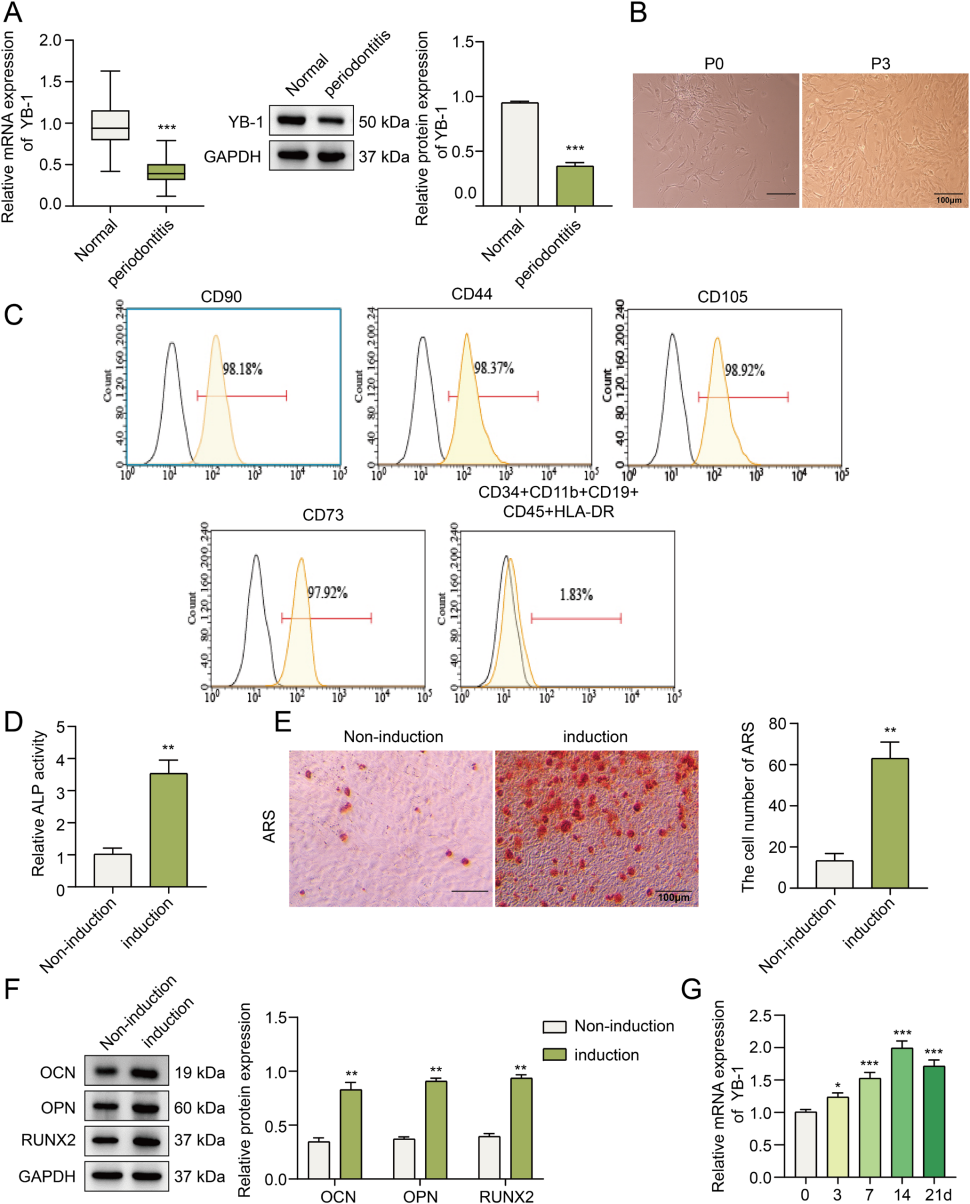
Expression of YB‐1 in the clinical tissues of periodontitis patients and hPDLSCs during osteogenic differentiation. The expression of YB‐1 at the mRNA and protein levels in the periodontal ligaments of patients with periodontitis was evaluated using qPCR and Western blotting (A), respectively. (B) The morphology of hPDLSCs was observed by microscopy. (C) Surface antigen markers of hPDLSCs were identified by flow cytometry. (D) Determination of ALP activity by a commercial kit. (E) Mineralized nodules were observed by ARS staining. (F) The protein expression of OCN, OPN and RUNX2 was analyzed using Western blotting. (G) The mRNA level of YB‐1 during the osteogenic differentiation of hPDLSCs was detected by qPCR. *n* = 3. *p < 0.05, **p < 0.01, ***p < 0.001.

To verify the role of YB‐1 in periodontitis, we collected periodontal ligament tissues from patients with clinical periodontitis and examined the differential expression of YB‐1. As shown in Figure 1A, the expression of YB‐1 at the mRNA and protein levels in periodontitis tissues clearly decreased compared with that in the normal group (Figure 1A). We subsequently identified the isolated human periodontal stem cells, and by microscopic examination, we observed under the microscope that the cell morphology was uniform with a slender spindle shape (Figure 1B). The flow cytometry results revealed that hPDLSCs positively expressed the mesenchymal‐cell‐like surface antigen markers CD44, CD90, CD73, and CD105 (positive cells > 97%) but negatively expressed the hematopoietic‐ and epithelial‐cell‐like specific antigen markers CD34, CD11b, CD19, CD45, and HLA‐DR (positive cells < 5%) (Figure 1C). These results revealed that hPDLSCs feature mesenchymal stem cells. In addition, *Porphyromonas gingivalis* lipopolysaccharide (p.g‐LPS), an important virulence factor in the mechanism of periodontal disease, led to a significant decrease in the expression level of YB‐1 in hPDLSCs (Figure S1). These findings suggest that the downregulation of YB‐1 might be related to bacterial infection and inflammation. Next, we induced hPDLSC osteogenic differentiation, and ALP activity was markedly greater in the osteogenic induction group than in the noninduction group (Figure 1D). Moreover, hPDLSCs produced more mineralized nodules after osteogenic induction (Figure 1E). In addition, the expression levels of OCN, OPN, and RUNX2 were markedly greater in the osteogenic induction group than in the noninduction group (Figure 1F). Intriguingly, we also confirmed gradually increased expression of YB‐1 during the osteogenic differentiation of hPDLSCs (Figure 1G). In summary, the dysregulation of YB‐1 expression was correlated with the osteogenic differentiation of hPDLSCs.

### 3.2 Knockdown of YB‐1 Suppresses the Proliferation and Osteogenic Differentiation of hPDLSCs

**FIGURE 2:**
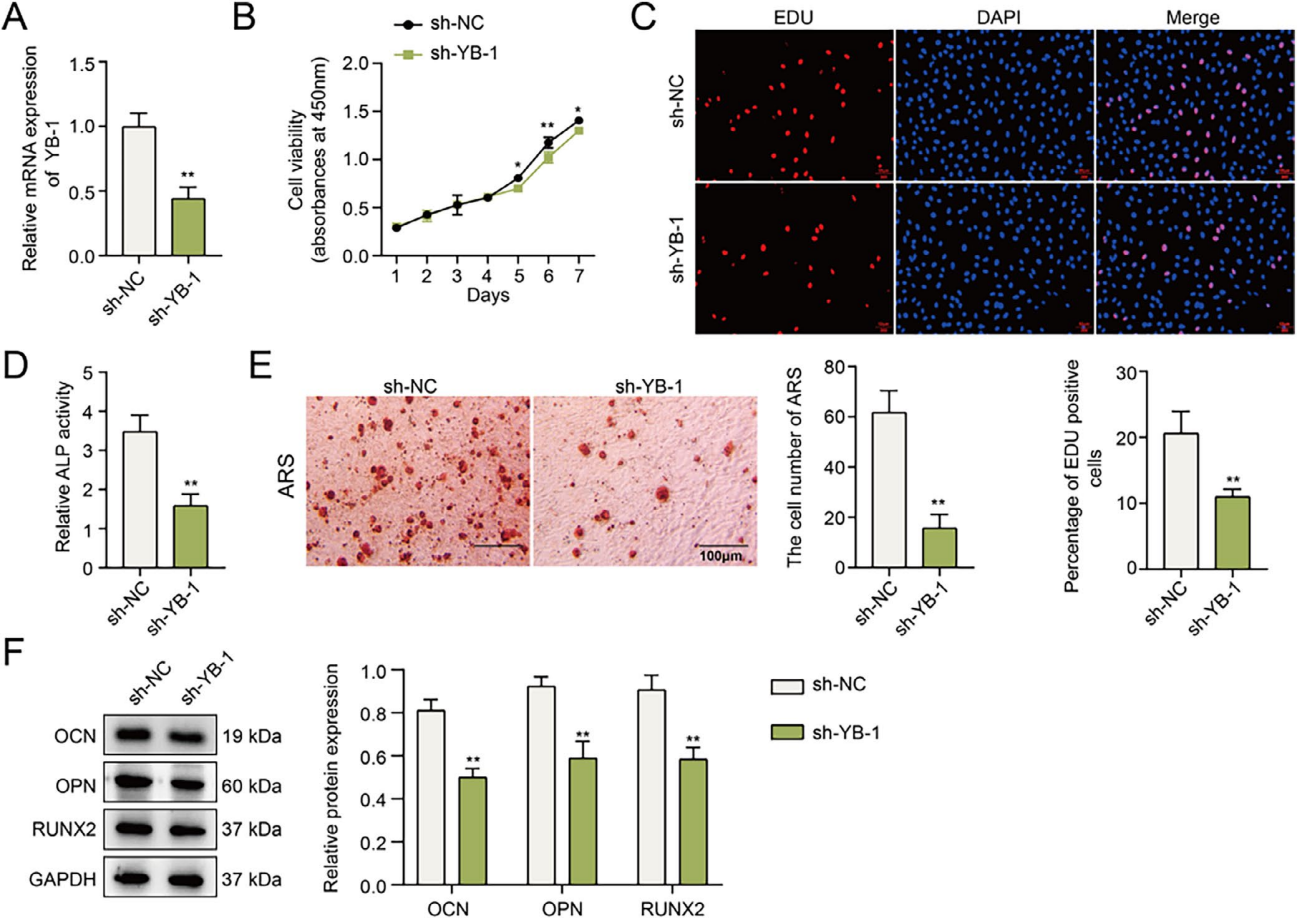
YB‐1 knockdown suppresses the proliferation and osteogenic differentiation of hPDLSCs. sh‐YB‐1 was transfected into hPDLSCs. (A) The knockdown efficiency was verified by qPCR. (B) The viability of hPDLSCs was detected by a CCK8 assay. (C) The proliferation of hPDLSCs was assessed using an EdU assay. (D) ALP activity in hPDLSCs was detected with a commercial kit after osteogenic induction. (E) After osteogenic induction, the mineralized nodules were observed by ARS staining. (F) The protein expression levels of OCN, OPN and RUNX2 were analyzed using Western blotting after osteogenic induction. *n* = 3. *p < 0.05, **p < 0.01, ***p < 0.001.

To evaluate the potential impact of YB‐1 on the proliferation and osteogenic differentiation of hPDLSCs, we altered the expression level of YB‐1 by knockdown with shRNA plasmids. Not surprisingly, the expression level of YB‐1 after sh‐YB‐1 transfection was markedly decreased (Figure 2A). Notably, the results of the CCK‐8 and EdU assays revealed that YB‐1 silencing markedly reduced cell viability and the number of EdU‐positive cells (Figure 2B,C). As shown in Figure 2D,E, knocking down YB‐1 significantly diminished ALP activity and mineralized nodules. Additionally, YB‐1 silencing decreased the protein expression of OCN, OPN, and RUNX2 (Figure 2F). Overall, YB‐1 plays essential roles in hPDLSC proliferation and osteogenic differentiation.

### 3.3 YB‐1 Promotes hPDLSC Proliferation and Osteogenic Differentiation by Regulating the Akt/GSK3β/β‐Catenin Signaling Pathway

**FIGURE 3:**
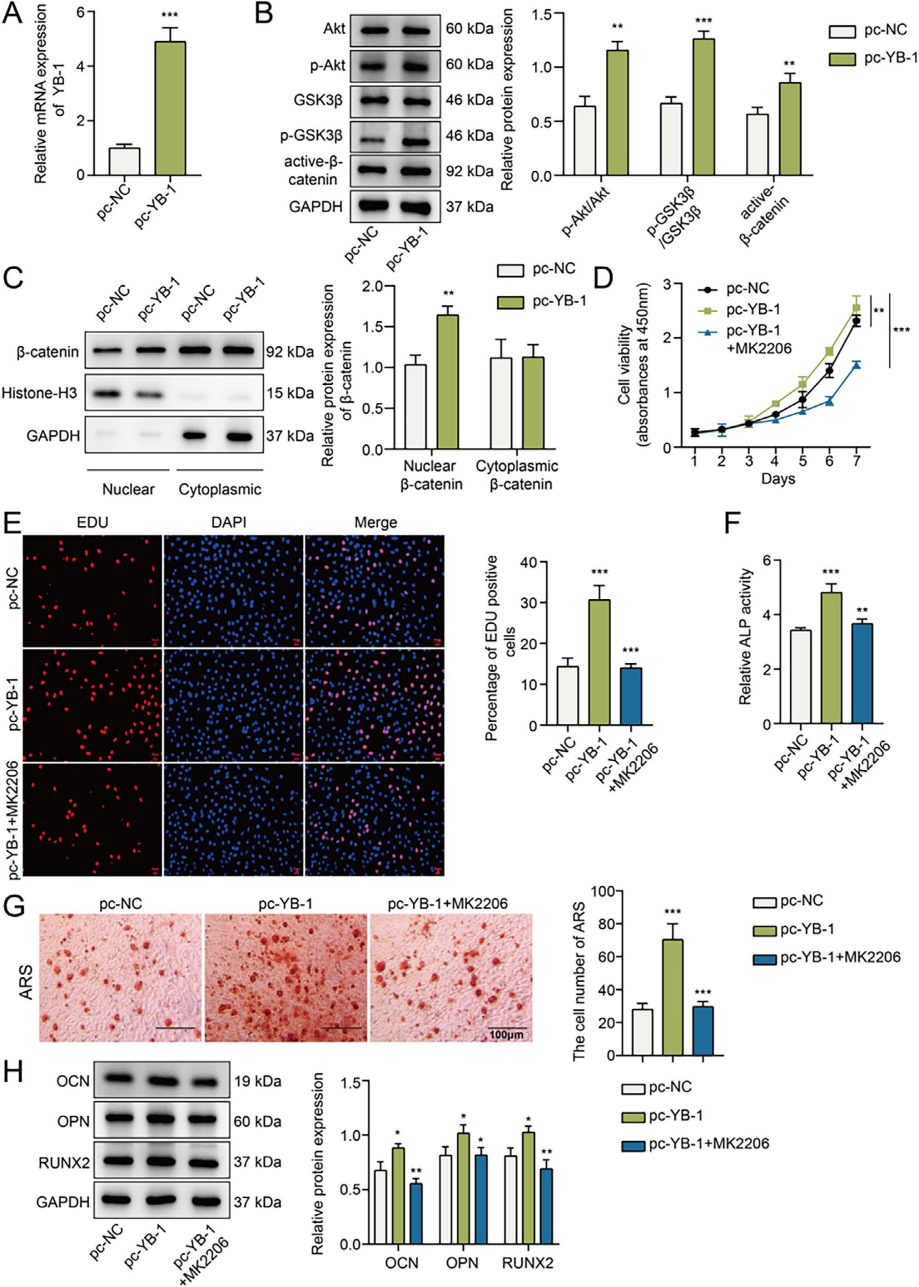
YB‐1 stimulates the proliferation and osteogenic differentiation of hPDLSCs by regulating the Akt/GSK3β/β‐catenin signaling pathway. pc‐YB‐1 was transfected into hPDLSCs. (A) The overexpression efficiency was verified by qPCR. (B) The protein levels of Akt/GSK3β/β‐catenin pathway‐related proteins were analyzed utilizing Western blotting. (C) The protein levels of β‐catenin in the nucleus and cytoplasm were analyzed utilizing Western blotting. hPDLSCs were treated with an Akt inhibitor after transfection with pc‐YB‐1. (D) hPDLSC viability was detected by a CCK8 assay. (E) The proliferation of hPDLSCs was assessed using an EdU assay. (F) ALP activity in hPDLSCs was detected by a commercial kit after osteogenic induction. (G) After osteogenic induction, the mineralized nodules were observed by ARS staining. (H) The protein expression levels of OCN, OPN and RUNX2 were analyzed utilizing Western blotting after osteogenic induction. *n* = 3. *p < 0.05, **p < 0.01, ***p < 0.001.

As previously stated, activating the AKT/GSK3β/β‐catenin pathway positively regulates the osteogenic differentiation of MSCs [13]. To determine whether the regulatory effect of YB‐1 on proliferation and osteogenic differentiation is related to the AKT/GSK3β/β‐catenin axis, hPDLSCs were subjected to pc‐YB‐1 transfection, and a qPCR assay was used to validate the efficiency of YB‐1 overexpression (Figure 3A). YB‐1 overexpression significantly weakened the promoting effect of p.g‐LPS on the levels of inflammatory factors (TNF‐α, IL‐1β and IL‐6) in hPDLSCs (Figure S1). Further evidence from Western blot assays revealed that YB‐1 overexpression increased the expression of p‐Akt/Akt, p‐GSK3β/GSK3β and active‐β‐catenin in hPDLSCs (Figure 3B). In addition, the overexpression of YB‐1 increased the protein expression of β‐catenin in the nucleus (Figure 3C). To clarify the regulatory role of the AKT/GSK3β/β‐catenin axis in YB‐1‐mediated hPDLSC osteogenic differentiation, hPDLSCs were pretreated with MK‐2206 to block the Akt pathway. MK‐2206 attenuated the influence of YB‐1 overexpression on the increased proliferation of hPDLSCs (Figure 3D,E). Additionally, the promoting effect of YB‐1 overexpression was partially abrogated by MK‐2206 (Figure 3F–H). In summary, we confirmed that the promoting effects of YB‐1 on hPDLSC proliferation and osteogenic differentiation were associated with activation of the Akt/GSK3β/β‐catenin signaling pathway.

### 3.4 YB‐1 Regulates the Akt/GSK3β/β‐Catenin Pathway in hPDLSCs Through the Transcriptional Activation of FGF2

**FIGURE 4:**
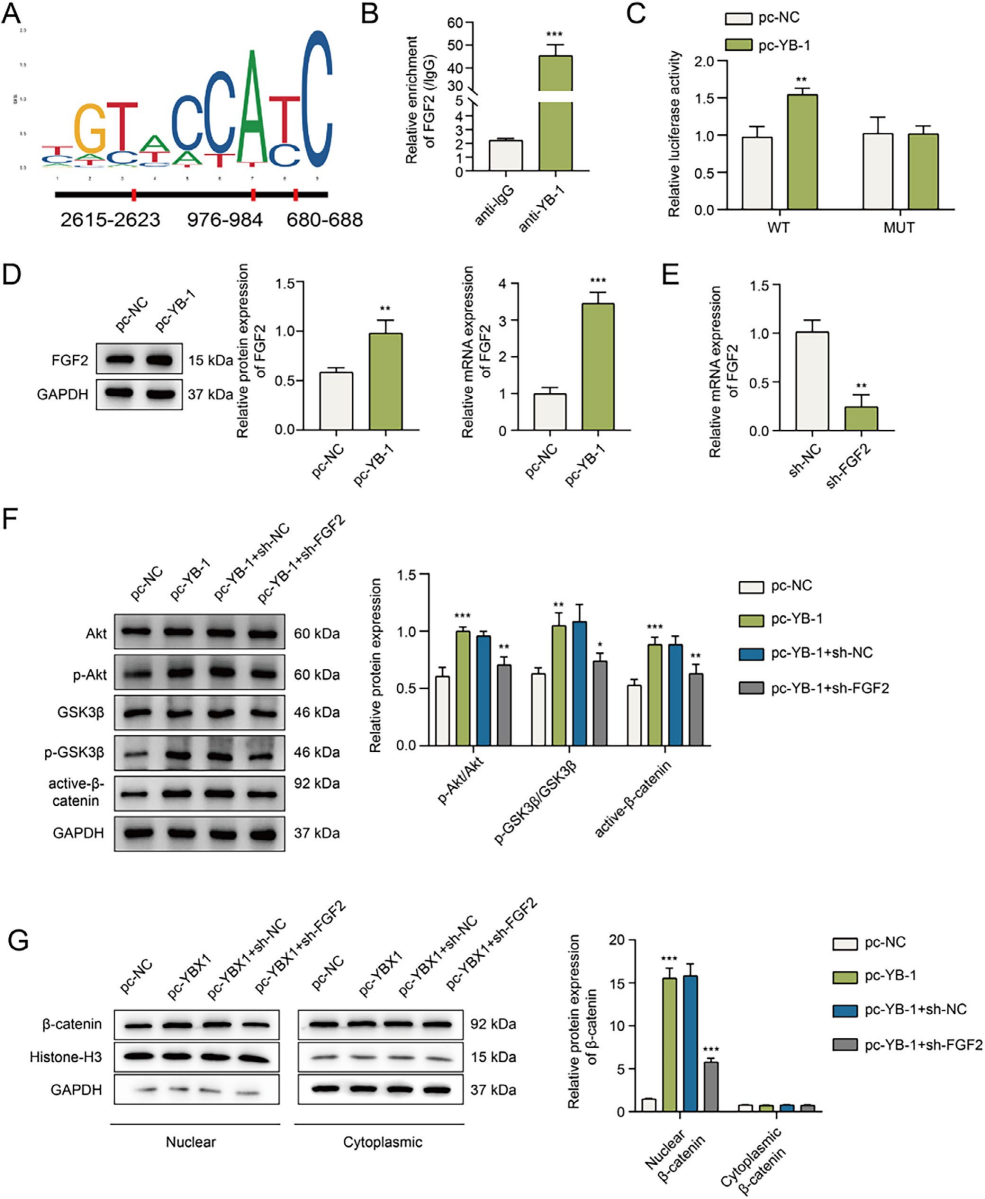
YB‐1 regulates Akt/GSK3β/β‐catenin signaling in PDLSCs through transcription to activate FGF2. (A) The binding sites between YB‐1 and the FGF2 promoter region were predicted through hTFtarget online analysis. (B) The interaction between YB‐1 and FGF2 was verified using a ChIP assay. (C) Luciferase activity at the YB‐1 and FGF2 sites was detected using a dual‐luciferase reporter gene assay. (D) FGF2 expression at the mRNA and protein levels was analyzed utilizing Western blotting after YB‐1 overexpression. (E) The knockdown efficiency of FGF2 was verified using qPCR after transfection with sh‐FGF2. (F) The protein levels of Akt/GSK3β/β‐catenin pathway‐related proteins were analyzed by Western blotting after simultaneous transfection with pc‐YB‐1 and sh‐FGF2. (G) The protein level of β‐catenin in the nucleus or cytoplasm was analyzed using Western blotting after simultaneous transfection with pc‐YB‐1 and sh‐FGF2. *n* = 3. *p < 0.05, **p < 0.01, ***p < 0.001.

A previous study suggested that FGF2 could affect the osteogenic differentiation of MSCs through regulating AKT signaling [24]. However, whether FGF2 regulates Akt/GSK3β/β‐catenin signaling to mediate hPDLSC osteogenic differentiation has not been reported. Through hTF target online analysis, we identified binding sites between the YB‐1 and FGF2 promoter regions (Figure 4A). Next, a ChIP assay confirmed the interaction between YB‐1 and FGF2, indicating that YB‐1 could bind to the FGF2 promoter region (Figure 4B). Moreover, a dual‐luciferase reporter assay revealed that YB‐1 overexpression apparently increased the luciferase activity of wt‐FGF2 but did not significantly affect mut‐FGF2 (Figure 4C). In addition, the overexpression of YB‐1 promoted the mRNA and protein expression of FGF2 (Figure 4D). Furthermore, hPDLSCs were successfully transfected with sh‐FGF2 (Figure 4E), and the phosphorylation levels of Akt and GSK3β as well as the protein level of active β‐catenin increased with the overexpression of YB‐1, whereas their levels were significantly decreased by knocking down FGF2 (Figure 4F). In addition, the amount of β‐catenin protein in the cell nucleus increased with the overexpression of YB‐1, while FGF2 knockdown reversed this increase (Figure 4G). Taken together, these results indicate that YB‐1 bound to FGF2 and transcriptionally regulated its expression, thereby activating the Akt/GSK3β/β‐catenin pathway in hPDLSCs.

### 3.5 FGF2 Reversed the Impact of YB‐1 Knockdown on the Osteogenic Differentiation and Proliferation of hPDLSCs

**FIGURE 5:**
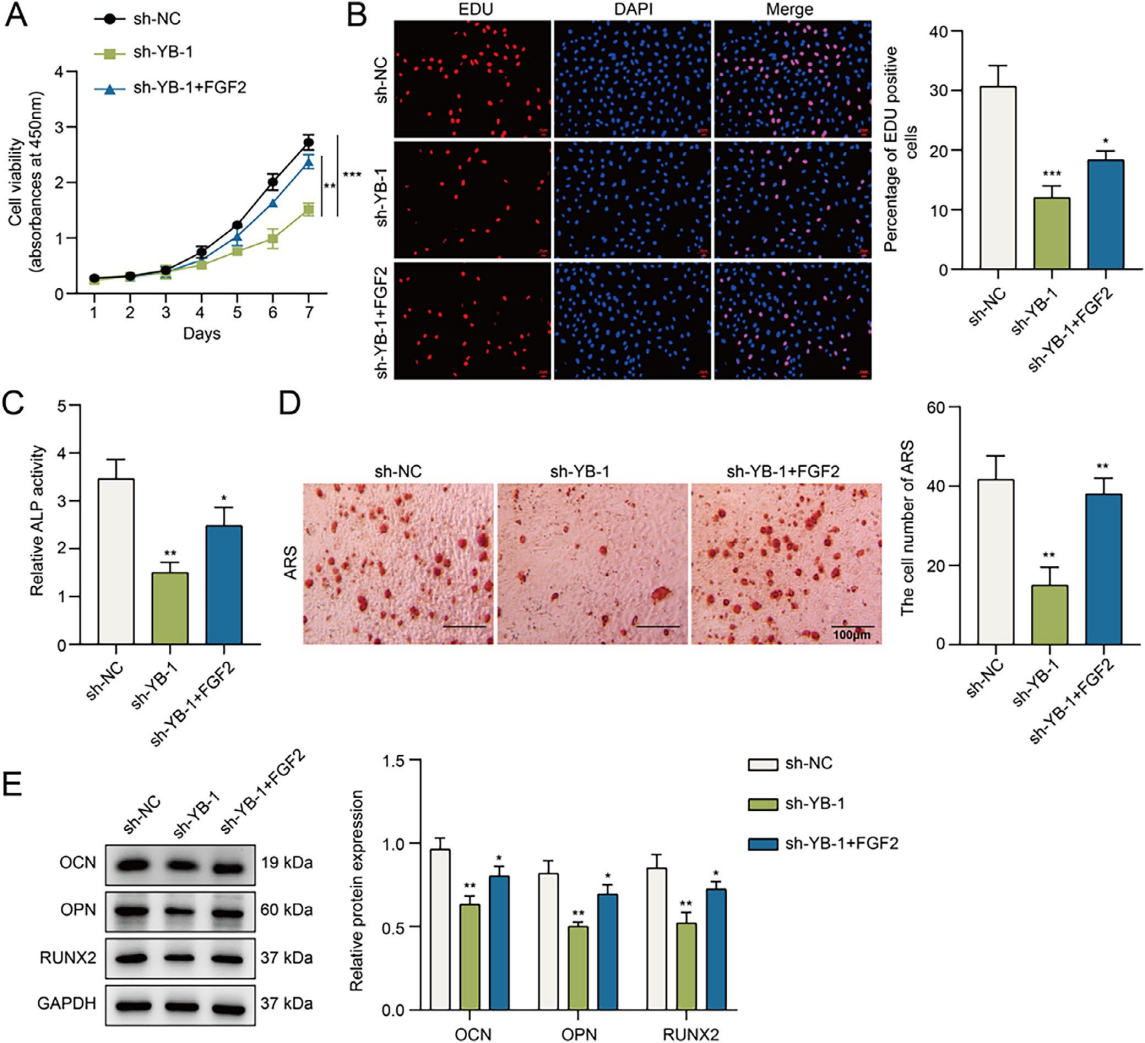
FGF2 reversed the impact of YB‐1 knockdown on the proliferation and osteogenic differentiation of hPDLSCs. hPDLSCs were transfected with sh‐YB‐1 prior to human recombinant FGF2 treatment. (A) The viability of hPDLSCs was detected via a CCK8 assay. (B) The proliferation of hPDLSCs was assessed using an EdU assay. (C) ALP activity in hPDLSCs was detected by a commercial kit after osteogenic induction. (D) After osteogenic induction, the mineralized nodules were observed by ARS staining. (E) The protein expression levels of OCN, OPN and RUNX2 were analyzed utilizing Western blotting after osteogenic induction. *n* = 3. *p < 0.05, **p < 0.01, ***p < 0.001.

To further verify whether YB‐1 affects the osteogenic differentiation of hPDLSCs through enhancing FGF2, hPDLSCs were transfected with sh‐YB‐1 prior to human recombinant FGF2 treatment. The results displayed that recombinant FGF2 treatment at 50 ng/mL significantly promoted cell proliferation (Figure S2), and the suppression effect of YB‐1 knockdown on cell proliferation was alleviated by FGF2 (Figure 5A,B). Additionally, the suppressive effect of YB‐1 knockdown on ALP activity and mineralized deposits was significantly reversed by FGF2 (Figure 5C,D). Furthermore, the protein levels of OCN, OPN, and RUNX2 were decreased by YB‐1 knockdown, while FGF2 abolished these effects (Figure 5E). On the basis of these results, we concluded that YB‐1 increased hPDLSC proliferation and osteogenic differentiation via the upregulation of FGF2.

## 4 Discussion

Periodontitis, which is caused mainly by gram‐negative bacterial infection, is the most common oral infectious disease and has serious adverse effects on the oral function, facial esthetics, and quality of life of patients [25]. Periodontal stem cells are a type of mesenchymal stem cell with multidifferentiation potential that can differentiate into osteoblasts, fibroblasts, and cementoblasts. Periodontal stem cells have the potential to regulate alveolar bone remodeling and are the preferred cells for studying periodontal tissue regeneration [26]. In this study, we successfully obtained and identified primary hPDLSCs and confirmed the osteogenic differentiation capacity of hPDLSCs by Alizarin red staining and ALP activity detection. Studies have shown that the osteogenic differentiation and proliferation of PDLSCs in periodontitis are significantly weakened, which is not conducive to the tissue repair role of PDLSCs in the treatment of periodontitis [27]. Therefore, studying the gene regulatory mechanism of PDLSC proliferation and osteogenic differentiation is vital to provide guidance for the repair of alveolar bone defects and the treatment of periodontitis.

Many investigations have demonstrated that YB‐1 is closely related to stem cell differentiation [28, 29]. On the one hand, YB‐1 promotes the differentiation of dental pulp stem cells (DPSCs) through increasing the inclusion of RUNX2 exon 5 [30]. On the other hand, YB‐1 positively regulates the osteogenic differentiation of MSCs [21]. Similarly, we also reported decreased YB‐1 expression in periodontitis clinical tissues and elevated YB‐1 expression during osteogenic differentiation of hPDLSCs. Additionally, knockdown of YB‐1 suppressed the proliferation and osteogenic differentiation of PDLSCs, which was characterized by decreased cell viability, cell proliferation, ALP activity, mineralized deposits, and osteogenic markers. These data suggest that YB‐1 might be a prospective target for the treatment of periodontitis.

The PI3K/Akt signaling pathway is widely implicated in cell proliferation, differentiation, metastasis, apoptosis, metabolism, and other important processes. Recent studies have also revealed that the increase in osteogenic differentiation of mesenchymal stem cells is closely related to the activation of the PI3K/Akt pathway [31]. On the one hand, inhibition of the PI3K/Akt pathway can decrease the expression levels of ALP, OCN, Osterix, and RUNX2, thus inhibiting osteoblast differentiation [32]. On the other hand, as one of the key regulatory targets of this pathway, Akt can significantly inhibit the phosphorylation of GSK‐3β, leading to its inactivation and reducing the degradation of β‐catenin, thereby mediating the nuclear translocation of β‐catenin to promote the downstream expression of genes related to cell proliferation and osteogenesis [11, 12]. Consistent with these results, in our study, YB‐1 was found to activate the Akt/GSK3β/β‐catenin signaling pathway in hPDLSCs. Notably, MK‐2206, which acts as an Akt inhibitor, weakens the promoting effect of YB‐1 overexpression on the osteogenic differentiation and proliferation of hPDLSCs. However, the mechanism by which YB‐1 regulates Akt/GSK3β/β‐catenin signaling is still unclear.

FGF2 is a multifunctional growth factor of the FGF family that is widely involved in the regulation of cell growth, development, and aging [33]. FGF2 not only directly participates in the regulation of osteogenic differentiation but also regulates bone metabolism in coordination with Wnt/β‐catenin, bone morphogenetic proteins (BMPs), MAPK, and other signaling pathways [34, 35]. Investigations have demonstrated that FGF2 can affect the osteogenic differentiation of mesenchymal stem cells by regulating AKT signaling [24]. Here, our findings revealed that YB‐1 acts as a transcription factor that can directly bind to the promoter of FGF2 and transcriptionally upregulate its expression. Additionally, FGF2 knockdown abrogated the activation of the Akt/GSK3β/β‐catenin signaling pathway caused by YB‐1 overexpression. FGF2 has been proven to inhibit periodontal inflammation [36], and the upregulation of FGF2 can increase the proliferation and osteogenic differentiation of PDLSCs [37, 38]. This finding is consistent with our results, as human recombinant FGF2 reversed the impact of YB‐1 knockdown on the suppression of the proliferation and osteogenic differentiation of PDLSCs.

**FIGURE 6:**
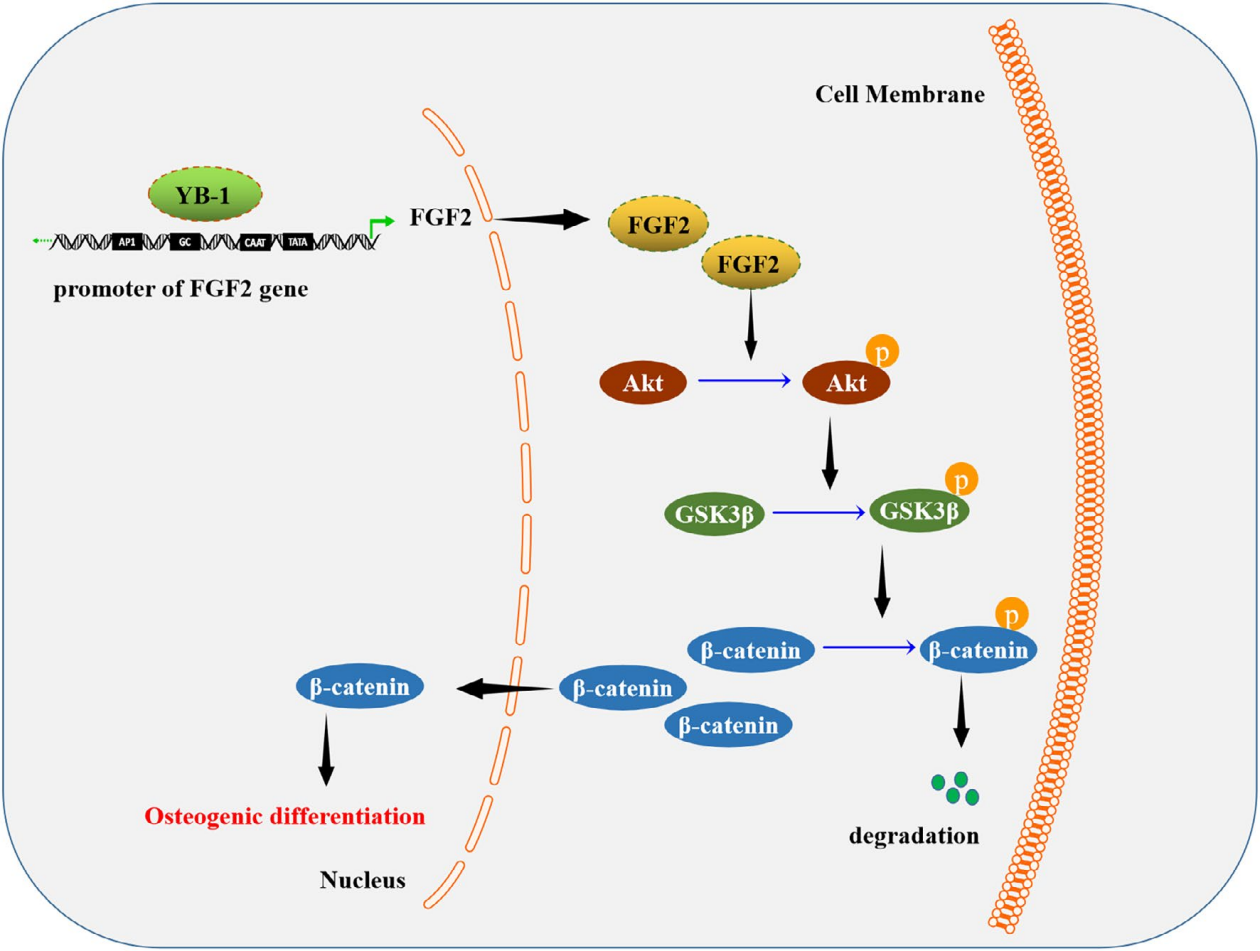
Illustration of graphic summary. YB‐1 overexpression increased FGF2 expression to activate the Akt/GSK3β/β‐catenin pathway, thereby promoting hPDLSC proliferation and osteogenic differentiation.

In conclusion, our findings indicated that YB‐1 overexpression increased FGF2 expression to activate the Akt/GSK3β/β‐catenin pathway, thereby promoting hPDLSC proliferation and osteogenic differentiation (Figure 6). Our findings provide new insights into the molecular mechanisms of YB‐1 in the proliferation and osteogenic differentiation of hPDLSCs and present further evidence for promising therapeutic targets in periodontitis.

## Ethics Statement

This study was approved by the Ethics Committee of Jiangsu Province Hospital on Integration Chinese and Western Medicine (No. 2024‐LWKY‐052).

## Conflicts of Interest

The authors declare no conflicts of interest.

## Supporting information


**Figure S1.** Overexpression of YB‐1 inhibited the levels of inflammatory factors in hPDLSCs induced by p.g‐LPS.
hPDLSCs were induced with 10 μg/mL p.g‐LPS. (A) The mRNA level of YB‐1 was detected via qPCR. (B) The levels of inflammatory factors were detected by ELISA. *n* = 3. *p < 0.05, **p < 0.01, ***p < 0.001.

**Figure S2.** Effects of human recombinant FGF2 on hPDLSC proliferation.
hPDLSCs were treated with different concentrations of recombinant FGF2. (A) Cell proliferation was evaluated by a CCK‐8 assay. *n* = 3. *p < 0.05, **p < 0.01, ***p < 0.001.

## Data Availability

All data generated or analysed during this study are included in this article. The datasets used and/or analysed during the current study are available from the corresponding author on reasonable request.
