# Supplementary figures and images for: Y‐Box‐Binding Protein 1 Facilitates the Proliferation and Osteogenic Differentiation of Periodontal Ligament Stem Cells Through the Transcriptional Activation of FGF2‐Mediated Akt/GSK3β/β‐Catenin Signaling
Source: Kaohsiung J Med Sci. 2025 Jul 30;41(12):e70079. doi: 10.1002/kjm2.70079 (PMC12694562; doi:10.1002/kjm2.70079)

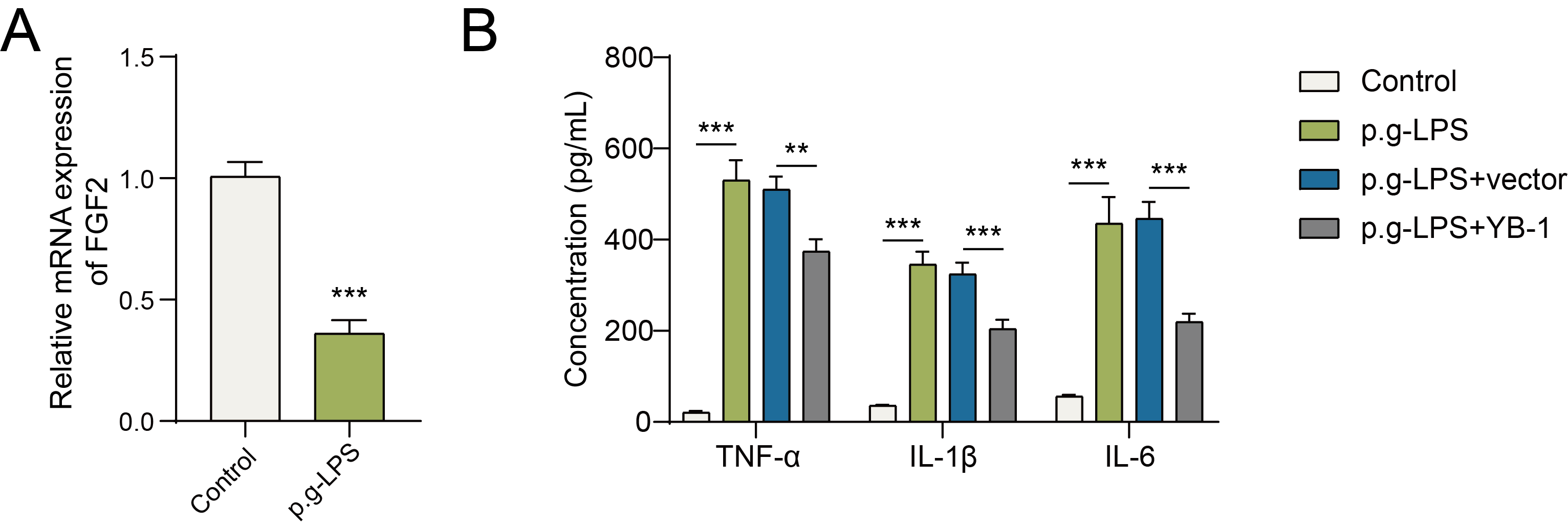

Supplement: Supplementary file 1 — Figure S1. Overexpression of YB‐1 inhibited the levels of inflammatory factors in hPDLSCs induced by p.g‐LPS. hPDLSCs were induced with 10 μg/mL p.g‐LPS. (A) The mRNA level of YB‐1 was detected via qPCR. (B) The levels of inflammatory factors were detected by ELISA. n = 3. *p < 0.05, **p < 0.01, ***p < 0.001. [file KJM2-41-e70079-s002.tif]

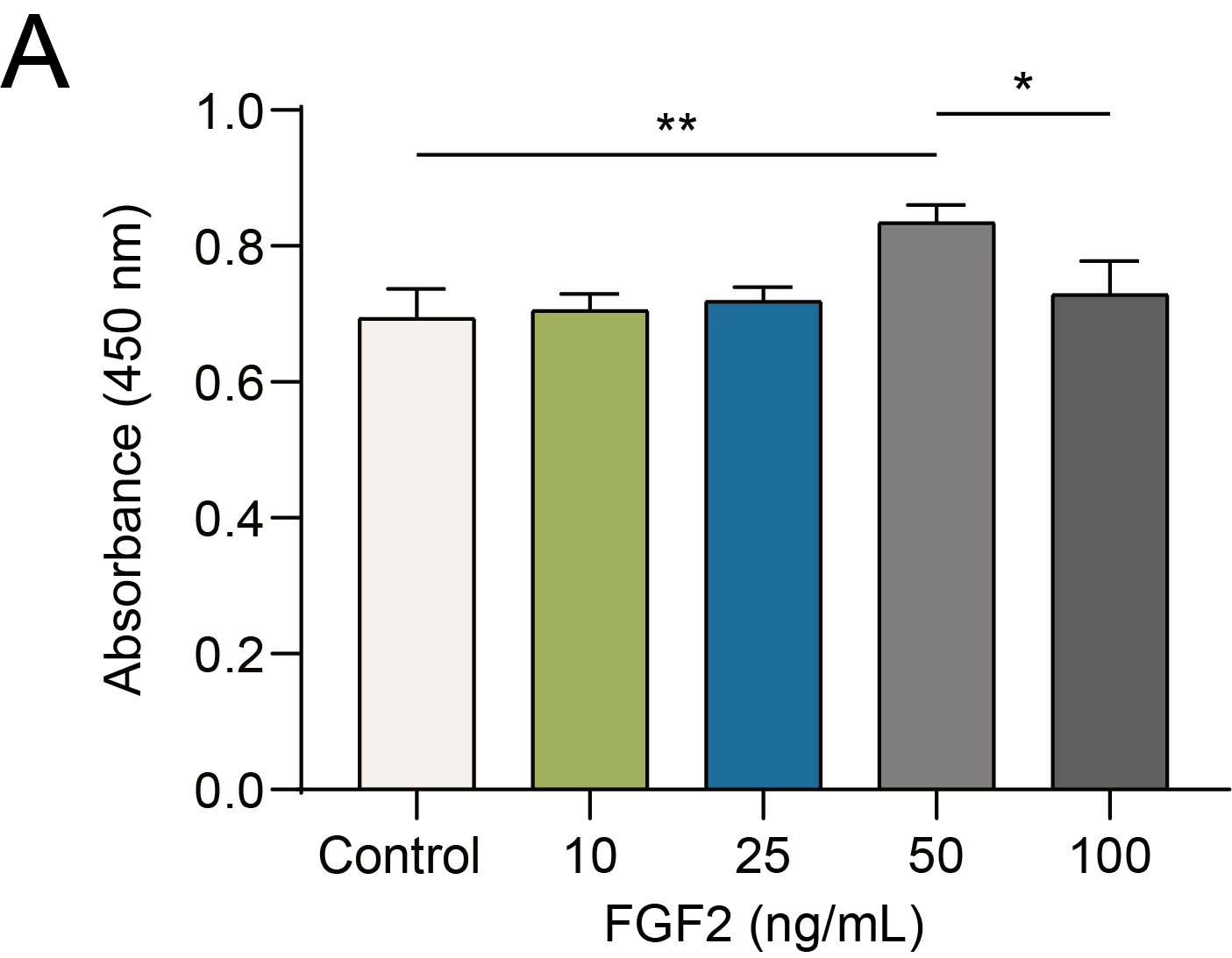

Supplement: Supplementary file 2 — Figure S2. Effects of human recombinant FGF2 on hPDLSC proliferation. hPDLSCs were treated with different concentrations of recombinant FGF2. (A) Cell proliferation was evaluated by a CCK‐8 assay. n = 3. *p < 0.05, **p < 0.01, ***p < 0.001. [file KJM2-41-e70079-s001.tif]
